# MCS Assisted Accurate Perception Framework for Urban POI Classification

**DOI:** 10.3390/s25237235

**Published:** 2025-11-27

**Authors:** Xiaorong Feng, Yuchen Yang, Xudong Zhang, Dongsheng Guo, Guisong Yang

**Affiliations:** 1 Engineering Training Center, Nantong University, Nantong 226019, China; fengxiaorong@ntu.edu.cn; 2 Department of Computer Science and Engineering, University of Shanghai for Science and Technology, Shanghai 200093, China; yuchen_yang2024@163.com (Y.Y.); 222320573@st.usst.edu.cn (X.Z.); 202580527@st.usst.edu.cn (D.G.)

**Keywords:** mobile crowd sensing, urban POI, participatory MCS, clustering algorithm

## Abstract

**Highlights:**

**What are the main findings?**

- Proposes a hybrid MCS perception framework (MCS-APF) that integrates participatory and opportunistic sensing to achieve high-quality, low-cost urban POI data collection.
- Introduces an improved DBSCAN-H clustering algorithm using Haversine distance for accurate geographic POI clustering, enhancing intra-cluster compactness and inter-cluster separation.

**What are the implications of the main finding?**

- Enables more efficient and accurate urban POI classification, supporting better decision-making in urban planning and sustainable development.
- Provides a scalable and cost-effective solution for large-scale urban sensing applications, with potential extensions to other spatial data analysis domains.

**Abstract:**

The classification of urban points of interest (POI) reflects the development of various industries in a city, making their distribution analysis significant. Traditional mapping methods often face inefficiency and high costs, leading to limited data quality and inaccuracies in classification. To address this, a low-cost, high-quality method is essential. Mobile Crowd Sensing (MCS) technology offers an innovative solution for identifying urban POIs. This paper introduces a hybrid MCS perception framework (MCS-APF) that includes a data collection module and a clustering module. The data collection module combines traditional participatory and opportunistic methods, incorporating a new recruitment criterion considering workers’ abilities, reputations, and POI popularity to enhance data quality. The clustering module employs an improved version of the Density-Based Spatial Clustering of Applications with Noise (DBSCAN-H) algorithm using Haversine distance, which effectively analyzes the combined data for accurate POI classification. Experimental results show that POI classifications derived from DBSCAN-H feature significant intra-cluster tightness and inter-cluster separation, outperforming traditional techniques. Overall, MCS-APF provides more accurate, efficient, and cost-effective POI sensing outcomes.

## 1. Introduction

In Geographic Information Systems, a point of interest (POI) can refer to locations such as houses, bus stops, and other features [1]. A POI represents a specific characteristic in an area that is often interesting or useful to people. The concept of POIs is widely used in fields like cartography and navigation. POI data typically includes information such as name, address, and category.

The POI data of an urban area can provide detailed insights into the development and changes occurring within that city. For instance, examining the distribution and development of POIs at a city’s tourist attractions can accurately reflect the overall growth of the tourism industry in that area. Understanding various types of POIs and their development patterns enables stakeholders to comprehend the status of different industries within the city. This knowledge is beneficial not only for industry practitioners but also for policymakers, as it aids them in formulating effective urban development plans.

Traditional methods of POI perception rely on professionals mapping out the data, which can achieve high accuracy and data completeness. Unfortunately, these methods come with high costs, long update periods, and poor timeliness.

To address the issues of cost, timeliness, and lengthy update cycles associated with traditional urban POI perception methods, there is a pressing need for a low-cost, rapid, and accurate approach. With advancements in internet technology and the widespread use of mobile devices, mobile crowd sensing (MCS) has emerged as a new data collection method that can facilitate urban POI perception. This approach utilizes a large number of users’ mobile devices as basic sensing units, combining conscious participation with unconscious collaboration. It allows for the distribution of sensing tasks and the collection of valuable data, effectively completing large-scale and complex sensing projects.

As a novel perception paradigm, MCS leverages ordinary users and their intelligent devices to complete sensing tasks. By deploying various sensors on these devices, large-scale applications can be efficiently constructed, significantly reducing the costs associated with specialized sensing infrastructure. Consequently, compared to traditional sensor networks, MCS has the characteristics of large scale, high flexibility, and low cost. On this basis, a large number of mobile swarm intelligence sensing platforms have emerged, such as Medusa [2], PRIS [3], WAZE [4], and CrowdOS [5]. In this trend, mobile group intelligence perception has become a crucial technological support for various fields, including intelligent transportation, urban management, and medical services.

In urban POI collection, MCS methods provide benefits in terms of both data quantity and quality. These methods are primarily divided into two approaches: participatory sensing and opportunistic sensing. Participatory sensing involves actively recruiting participants to upload photos, record videos, or complete questionnaires. This approach allows for the flexibility and diversity of participant contributions, enabling a wide range of data types to be collected. However, challenges include the high costs associated with recruitment and management, as well as potential limitations in participant quality, which can impact the volume and accuracy of the POI distribution analysis.

Opportunistic sensing, on the other hand, strategically leverages participants’ daily activities to collect information without requiring any extra time or effort. For instance, mobile devices and social media can record geolocation and environmental data. While this approach takes advantage of participants’ free time, it faces several significant challenges such as a lack of detailed information on POI, the risk of malicious or duplicate data, and difficulties in ensuring consistent data quality.

While MCS offers a promising alternative, the inherent limitations of both participatory and opportunistic sensing impede its ability to efficiently achieve high-precision POI perception. Consequently, a significant research gap exists in effectively integrating these two paradigms to leverage their strengths—ensuring data quality from participatory sensing while expanding data coverage at a low cost through opportunistic sensing.

This paper proposes an MCS-assisted accurate perception framework for urban POI classification. The specific working process of the method is as follows: firstly, the sensing platform obtains the data related to urban POI through two ways: participatory sensing and opportunistic sensing. In participatory sensing, a data quality assurance worker recruitment algorithm is proposed in this paper to address the challenge of how the sensing platform can recruit workers who can provide high-quality sensing data with a limited budget. The proposed POI spatiotemporal requirement criteria are used in conjunction with genetic algorithms for optimization to recruit a set of workers that can maximize the quality of perceptual data while ensuring that the cost to the platform remains limited. This approach ensures the collection of high-quality urban POI data. Subsequently, the high-quality participatory sensing data is fused and processed with the content-rich, large-volume, and low-cost opportunistic sensing data. An improved DBSCAN clustering algorithm based on Haversine’s distance formula is then proposed to cluster and analyze the fused data, to finally obtain the distribution of urban POI.

The main contributions of this paper are as summarized as follows:

1. In the participatory perception mode, a high-quality worker recruitment algorithm based on a genetic algorithm named WR-GA is designed to recruit high-quality workers to perform perception tasks, collect high-quality POI data, and ensure high accuracy.
2. An improved DBSCAN algorithm named DBSCAN-H is designed to represent the actual distance between position sample points through Haversine distance to ensure the accuracy of clustering results.

The remainder of the paper is structured as follows. Section 2 provides a summary of existing related studies. Section 3 presents the proposed system model and problem definition. Section 4 discusses the mechanism and optimization model of the data collection module and clustering module. Section 5 presents the performance evaluation, which analyzes the simulation results. Finally, Section 6 presents the conclusions.

## 2. Related Works

### 2.1. POI Perception

The main research goal of POI perception is to analyze user behavior to perceive the distribution of POI in a city by using existing urban big data based on geolocation information. In recent years, several researchers have studied POI perception. In [6], the authors propose an unsupervised approach to extract representative views and images of each landmark using the photo data uploaded by multimedia users to realize the perception of urban POI. The authors of [7] propose a novel clustering algorithm P-DBSCAN, based on density-based DBSCAN, to cluster and analyze a large number of photos with geographical locations to discover POI. The authors of [8,9] propose an improved adaptive spectral clustering method by extracting heterogeneous features from images containing locations to achieve POI perception. The authors of [10] propose a probabilistic access POI identification method that uses a novel hierarchical Bayesian model to analyze the user’s movement actively to derive the user’s preferred POI. The authors of [11] use microblog check-in data to achieve a perceptual update of urban POI by matching these data spatially as well as by attributes. To uncover unpopular POI with potentially valuable value, the authors of [12] propose a hierarchical multi-cue fusion (HMCF) approach that uses user-generated content from multiple sources to comprehensively describe POI and finally finds potential POI by modeling them hierarchically. To address the problem of inferring the location of traffic violation hotspots in cities, the authors of [13] propose a framework based on MCS to dynamically infer traffic violation hotspots, which extracts locations with traffic violation tendencies from heterogeneous crowd-sensing data and utilizes a spatiotemporal context-aware adaptive learning model (CSTA) to infer traffic violation hotspots. The authors of [14] extracted spatiotemporal data from photos labeled with geographic information and recommended POI based on geographic labels. The authors of [15] propose a recommendation algorithm based on user similarity, POI popularity, and time context, which addresses the problem of decreased user decision-making efficiency. Zhang et al. demonstrate that traditional data fusion approaches often fail to account for temporal dynamics in urban POI data, leading to outdated perceptions in rapidly changing environments [15]. Similarly, Li et al. show that deep-learning-based POI classification models require extensive labeled datasets, which are rarely available in real-world MCS scenarios [16]. Current research shows that POI perception faces the following problems and challenges: firstly, the sparseness and incompleteness of data limit the accuracy and reliability of POI perception; secondly, existing methods face the challenges of computational resources and time cost when dealing with large data, and it is difficult to respond and adapt quickly to dynamic changes. Therefore, the development of POI-aware frameworks that can efficiently acquire and process large-scale data with good scalability is an urgent problem to be solved.

### 2.2. Mobile Crowd Sensing

Ensuring the quality of perceptual data is a key issue and a hot research topic in MCS. In the study of task assignment and worker recruitment, the authors of [17] view a quality-aware user recruitment problem as an optimization problem and analyze the correlation between data and contextual information through federated learning to predict the quality of perceptual data from different users. In [18], the authors propose a new multitasking framework, MTasker, which redefines the multitasking problem by introducing a minimum perceived quality threshold for a specific task to assign the appropriate set of tasks to each worker, thus maximizing the utility of the overall system. As for the research on incentive mechanisms, understanding how to motivate more users to participate in perceptual tasks and provide high-quality data has become one of the hot issues in MCS research. The authors of [19] propose an effective and quality-aware incentive mechanism through which participants are motivated to contribute perceptual data to maximize the amount of high-quality perceptual data with a limited task budget. In [20], the authors propose an incentive mechanism framework called Thanos that incorporates a key metric called worker information quality and uses a technique based on reverse portfolio auction to achieve high-quality data at little cost. In [21], the authors similarly use a reverse auction-based incentive mechanism framework to recruit workers who can provide high-quality perceptual data with minimal total worker compensation. Recent advancements continue to refine these aspects, particularly through the application of advanced deep learning and multi-agent systems. For instance, to address the complex interplay between platform profit and worker benefits, Zhang et al. [22] proposed MARCS, a framework utilizing multi-agent deep reinforcement learning (MADRL) integrated with Shapley value to fairly evaluate and reward individual data contributions. This approach effectively balances the interests of the platform with those of individual workers in dynamic environments. For the core task allocation problem, which is often NP-hard, traditional heuristic methods often suffer from performance loss. To this end, Li et al. [23] introduced an intelligent task allocation model that combines Graph Attention Networks (GAT) with Deep Reinforcement Learning (DRL). This hybrid model captures complex spatial-temporal dependencies among tasks and workers, demonstrating strong adaptive capabilities and outperforming traditional greedy or heuristic algorithms in large-scale scenarios. Furthermore, in dynamic scheduling scenarios requiring fine-grained control, Zhao et al. [24] developed a task partitioning and scheduling strategy based on the Twin Delayed Deep Deterministic Policy Gradient (TD3) algorithm. Their method effectively handles discrete action decisions in MCS, significantly reducing overall task completion latency and enhancing system responsiveness. The extant research demonstrates that MCS presents the following problems and challenges: firstly, the existing research primarily concentrates on the assessment of data quality in a single or limited number of dimensions, thereby failing to give due consideration to the multiple factors that affect data quality; secondly, the question of how to adjust the task allocation strategy in real-time to enhance system flexibility and response speed in dynamic and complex environments remains unanswered. It is therefore imperative to develop multi-objective recruitment criteria for worker recruitment strategies based on practical application scenarios, to obtain cost-effective perceptual data.

### 2.3. Clustering Algorithm

At present, the majority of clustering algorithms are frequently inadequate when confronted with clusters of disparate shapes and densities. The density-based clustering algorithm DBSCAN [25,26] defines the core object through the concept of density reachability. When the appropriate radius parameter Eps and the number of samples Minpts are selected, DBSCAN is capable of discovering clusters of varying shapes. Nevertheless, the selection of appropriate parameters remains a challenging task. To address this issue, the authors of [27] put forth the Density Peaks Clustering (DPC) algorithm. In comparison to DBSCAN, the parameters of DPC are more straightforward to ascertain and yield superior clustering accuracy. However, its computation based on a distance matrix does not exhibit a notable enhancement in terms of scalability. Subsequently, numerous studies have proposed enhanced schemes based on DPC. For instance, the CFSFDP-HD [28] algorithm employs a non-parametric density estimation method to optimize the local density; the DPCG [29] algorithm accelerates computation by utilizing grid objects instead of data objects. The VDPC [30] algorithm introduces a novel variational density peak clustering method. This method addresses the challenge of identifying variations in cluster densities. It performs the clustering task systematically and autonomously. It is particularly suited to datasets that exhibit diverse density distributions. The DPC-NNMS [31] algorithm presents a novel approach to density peak clustering, based on a natural neighborhood merging strategy. This method adaptively identifies the local density of each data point by determining its natural set of neighbors. The current research demonstrates that clustering algorithms present several challenges. Firstly, the scalability of the algorithms is a significant challenge. While some clustering algorithms are relatively simple in parameter selection, the calculation based on the distance matrix is less efficient when dealing with large-scale datasets [32]. Furthermore, it is difficult to expand these algorithms to big data scenarios. Secondly, the diversity of cluster shapes and densities presents a further challenge. Different algorithms have different performance when dealing with clusters of different shapes and densities. Furthermore, the large amount of noise and outliers in the actual data significantly affects the clustering effect. Consequently, the development of an efficient and accurate clustering algorithm for POI-aware real-world scenarios, as well as the fused data acquired in MCS, represents a significant challenge for MCS-APF.

To systematically summarize the limitations of existing works and visually highlight the research gap, we provide a comparative analysis in Table 1. The table clearly shows that while existing methods have made progress in specific areas, they often excel in one aspect at the expense of others, particularly struggling with the trilemma of balancing data quality, cost, and clustering accuracy. As visualized in the table, our proposed MCS-APF framework is designed to address these intertwined challenges holistically, which constitutes the primary target and contribution of this work.

## 3. System Model

**Figure 1:**
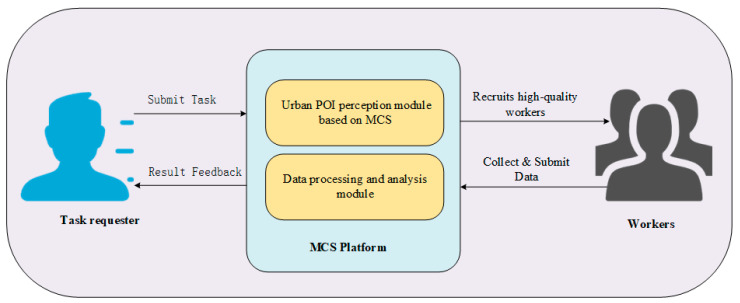
Urban POI perception model.

**Figure 2:**
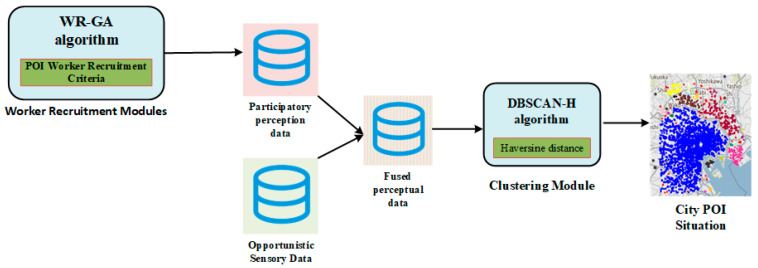
Urban POI sensing process based on fused data.

The MCS-assisted accurate perception framework for urban POI classification (MCS-APF) proposed in this paper aims to address the limitations of traditional POI perception (high cost, low timeliness) by integrating participatory MCS (for high-quality data) and opportunistic MCS (for large-scale coverage), and finally achieving accurate POI clustering via the improved DBSCAN-H algorithm. The system model clarifies the roles of stakeholders, data flow, and core functional modules, with its overall workflow and detailed perception process shown in Figure 1 and Figure 2, respectively.

### 3.1. Stakeholders and Role Definition

The system involves three core stakeholders, whose interactions directly determine the efficiency and accuracy of POI perception.

**Task Requester**: Mainly includes urban planning departments, navigation service providers, and industry researchers. They submit POI perception tasks to the perception platform, specifying key requirements such as the target geographic scope (e.g., the central area of Tokyo with latitude ranging from 35.55° N to 35.85° N and longitude from 139.60° E to 139.90° E), target POI categories (e.g., catering, transportation hubs, and shopping malls), and budget constraints (e.g., maximum worker recruitment costs not exceeding $5000).

**Perception Platform:** As the core hub of the entire framework, it undertakes three key functions. First, it recruits high-quality workers for participatory sensing using the WR-GA algorithm (Worker Recruitment based on Genetic Algorithm) (detailed in Section 4.1). This algorithm evaluates workers from three quantifiable dimensions—individual ability (historical accuracy of submitted POI data), reputation (compliance with task requirements such as no duplicate submissions), and POI popularity adaptability (experience in collecting data for specific POI types)—to ensure high-quality data collection within a limited budget. Second, it collects opportunistic sensing data, including public GPS trajectory data from mobile applications, geotagged photos from social media platforms, and environmental sensor data (e.g., Wi-Fi signal strength) from smart devices, to expand data coverage at a low cost. Third, it performs data fusion and clustering analysis: integrating multi-source data through spatial correction and temporal alignment, and inputting the fused data into the DBSCAN-H algorithm for clustering to obtain the final POI distribution, which is then fed back to the task requester.

**Workers**: Participants in participatory sensing, consisting of both professional users (e.g., geographic surveyors) and ordinary citizens. Their data quality directly determines the reliability of the subsequent fusion and clustering results. The platform selects workers based on the WR-GA algorithm to ensure that the collected data meets the accuracy requirements for POI perception.

### 3.2. Overall Workflow of MCS-APF

The workflow of MCS-APF forms a closed loop from task initiation to result feedback, with six sequential steps as shown in Figure 1:

**Task Submission**: The task requester sends a formal POI perception request to the perception platform, clearly stating the target area, POI category requirements, and budget limits. These requirements (e.g., target POI types, cost caps) are transmitted to the platform’s worker recruitment module as input parameters for the WR-GA algorithm.

**Participatory Worker Recruitment (Driven by WR-GA Algorithm)**: The perception platform invokes the WR-GA algorithm to screen potential workers from the candidate pool. By optimizing the trade-off between data quality and recruitment costs, it selects a set of workers who can provide high-quality data within the budget and issues data collection tasks to them (e.g., requiring submission of POI location coordinates, textual descriptions, and check-in times).

**High-Quality Data Collection**: Recruited workers use mobile devices to collect POI-related data, including user ID (${ID}_{i}$), check-in location ($L_{i}$ = ($x_{i}$, $y_{i}$), where *x_i_* represents longitude and *y_i_* represents latitude), check-in time ($T_{i}$), and textual description of the check-in location ($D_{i}$, e.g., “XX Restaurant, 2nd floor of XX Shopping Mall”). The platform verifies the completeness and validity of the submitted data in real time, rejecting submissions with missing key information (such as unrecorded latitude and longitude) or obvious errors (such as coordinates outside the target area).

**Opportunistic Data Acquisition**: To make up for the limited coverage of participatory sensing data, the platform collects opportunistic sensing data from non-recruited users’ daily activities. This type of data has the characteristics of large volume and low cost but may contain defects such as GPS drift (average error of 5–10 m in urban canyons), duplicate records, and lack of textual descriptions, which require subsequent processing.

**Data Fusion and Clustering Analysis**: The platform first preprocesses the two types of data. For spatial correction, it corrects the GPS drift of opportunistic sensing data using high-quality participatory sensing data as a reference. The 50-m threshold was determined based on typical urban GPS accuracy and existing literature. Studies [13] on urban positioning and our preliminary analysis of the Foursquare dataset indicated that over 95% of spurious GPS points in opportunistic data fall within a 50-m radius of the true location when referenced against high-accuracy participatory data. If the Haversine distance to the nearest participatory sensing point is less than 50 m, the coordinates of the opportunistic point are adjusted to match the participatory point. This threshold effectively filters out significant drift while retaining valuable nearby data points. For temporal alignment, it retains only data within the same 1-h window as the participatory sensing check-in time to reduce temporal redundancy. This window reduces temporal redundancy while preserving the semantic coherence of POI visits. For semantic supplement, it assigns the textual description of the nearest participatory sensing point to opportunistic points without semantic information to improve the accuracy of subsequent category labeling. The fused data is then input into the DBSCAN-H algorithm. This algorithm uses Haversine distance to calculate the actual geographic distance between points, avoiding the inaccuracy of Euclidean distance in geospatial data processing, and thus accurately grouping spatially adjacent POIs.

**Result Feedback**: The platform generates the final POI set ${POI}_{K}=\{{POI}_{1},{POI}_{2},\ldots ,{POI}_{i},\ldots ,{POI}_{k}\}$ based on the clustering results. Each ${POI}_{i}$ is represented as $\left\{\lambda_{i},\vartheta_{i},c_{i}\right\}$, where $\lambda_{i}$ and $\vartheta_{i}$ are the longitude and latitude of the POI cluster center (calculated as the average of the coordinates of all points in the cluster), and $c_{i}$ is the POI category (determined by majority voting on the textual descriptions of the points in the cluster). The platform then feeds this POI set back to the task requester to support urban planning, navigation services, and other application scenarios.

The workflow of the precise urban POI perception model based on MCS in this paper is as follows: Firstly, the task requester requests the task of precise urban POI perception from the perception platform. After receiving the task request, the perception platform recruits high-quality workers through a participatory perception mode to submit high-quality POI related data to ensure the accuracy of urban POI perception; However, due to the limited budget of the perception platform, only a small amount of high-quality data can be obtained. Then the perception platform uses the opportunistic perception mode to collect opportunistic city POI perception data with a large amount of data, rich content, and low cost. Finally, the perception platform integrates and analyzes the collected perception data to obtain the precise distribution of urban POI and provides feedback to the task requester. Figure 1 shows the process of working on the system model in this paper.

### 3.3. Urban POI Perception Based on Mobile Sensing Data

This subsection will introduce the urban POI perception module based on multi-source data. The main function of this module is to fuse high-quality participatory perception data with rich content, large data volume, and low-cost opportunistic perception data, and achieve perception of urban POI distribution through clustering analysis.

In this paper, a given user set of size *N* is $U_{N}=\{u_{1},u_{2},\ldots ,u_{i},\ldots ,u_{N}\}$. There are M types of interest points recorded as $C_{M}=\{c_{1},c_{2},\ldots ,c_{j},\ldots ,c_{M}\}$. We assume that the urban POI information collected based on Opportunistic Perception and Participatory Perception is mainly composed of the following parts: user ID, user check-in location, user check-in time, and user’s description of the check-in location. For a certain user $u_{i}$, the check-in information provided by the user includes: user ID ${ID}_{i}$, user check-in location $L_{i}$, user check-in time $T_{i}$, and the user’s description of the check-in location $D_{i}$. The user’s check-in location is composed of the longitude and latitude of the location, so $L_{i}=(x_{i},y_{i})$, where $x_{i}$ represents the longitude of the location, and $y_{i}$ represents the latitude of the location. To sum up, the check-in information of user $U_{i}$ can be represented by a five-tuple, namely {${ID}_{i},x_{i},y_{i},T_{i},D_{i}$}. The goal of this model is to analyze all user check-in information quintuples through clustering technology, and finally get K POI sets in the city, which are recorded as ${POI}_{K}=\{{POI}_{1},{POI}_{2},\ldots ,{POI}_{i},\ldots ,{POI}_{k}\}$. The analysis results of the model include the position and category of each POI. For ${POI}_{i}$, ${POI}_{i}=\{\lambda_{i},\vartheta_{i},c_{i}\}$, where $\lambda_{i}$ represents the longitude of the POI, $\vartheta_{i}$ represents the latitude of the POI, and $c_{i}\in C_{M}$ represents the category to which the POI belongs. However, due to many defects in the data provided by Opportunistic Perception Users, the urban POI distribution obtained at this time is often not accurate enough. The specific workflow of the model is shown in Figure 2.

## 4. Methods of MCS-APF

### 4.1. WR-GA Based on POI Quality

**Figure 3:**
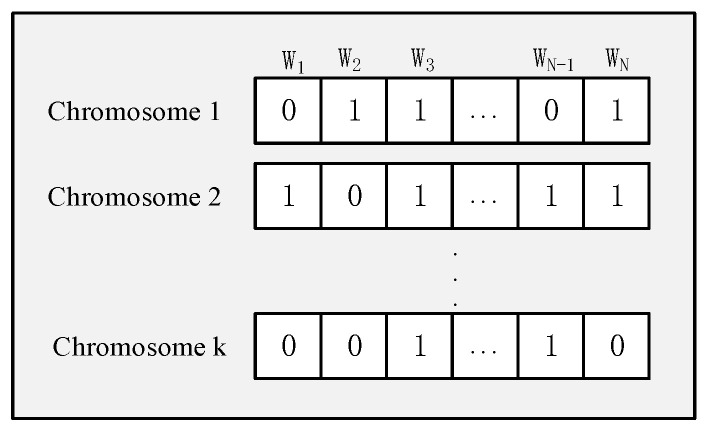
Coding.

The objective of the data collection module in the MCS-APF is to maximize the value for money of the data obtained by the platform by recruiting high-quality workers who meet the POI characteristics within a limited budget. This was done to ensure the quality and accuracy of the POI classification. A combination of platform budget, worker attributes, and POI attributes must be considered to select the workers who can maximize the POI-aware data to achieve this goal. Consequently, in this paper, we use the genetic algorithm, an efficient combinatorial optimization algorithm that mimics natural evolution. The key steps in the genetic algorithm are given in the following:

**Coding**: Coding is the process of transforming problem parameters that cannot be addressed directly by the genetic algorithm into chromosomes or individuals. In this paper, we must select high-quality workers from a pool of N workers, which aligns with the scope of application of binary coding, where 0 represents unselected and 1 represents selected. Figure 3 shows the binary code used in this paper. Each chromosome represents a solution. In chromosome 1, the gene value of $w_{1},w_{N-1}$ position is 0, indicating that they are not selected to perform the task, and the gene value of the $w_{2},w_{3},w_{N}$ positions is 1, indicating that they are selected to perform the task.

**Fitness function**: In genetic algorithms, the optimization objective is mapped to the chromosome fitness by the fitness function, and the optimization objective is achieved by utilizing the superiority of the chromosome. The objective of this paper is to maximize the total quality of the urban POI data $Q_{total}$ collected by the sensor platform. Consequently, the objective is directly employed as the fitness function of the genetic algorithm. In mathematical notation, it is expressed as $fitness=Q_{Total}$.

**Population initialization**: The objective of population initialization is to generate multiple sets of feasible solutions in a large solution space, thereby providing a basis for selection, crossover, and mutation operators. To ensure that the feasible solutions are uniformly distributed, this paper employs the random population initialization method, which randomly sets the gene of worker $w_{i}$ to 0 or 1 for each chromosome, and repeats this operation until a population containing N chromosomes is generated.

**Selection operator**: In genetic algorithms, common selection operations include sort selection, random league selection, roulette selection, etc. This paper will use the method of roulette to realize the selection operation. The specific operation process of roulette is as follows:

1. Calculate the fitness of each individual in the group and the fitness of the i-th individual is recorded as fitness(i)
2. Calculate the probability $p(i)=\frac{fitness(i)}{\sum_{j=1}^{N}fitness(j)}$ of the i-th individual being inherited by the next generation.
3. Calculate the cumulative probability $q(i)=\sum_{j=1}^{i}p(i)$ of the i-th individual.

**Figure 4:**
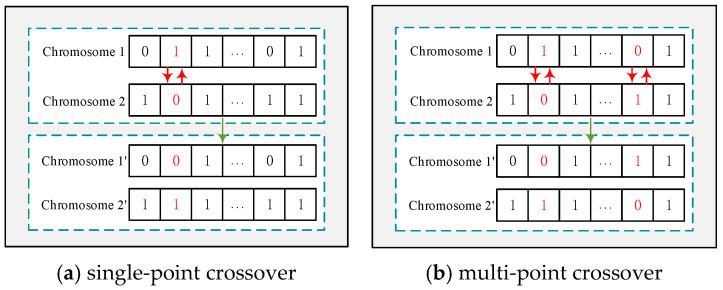
Crossover.

**Crossover operator**: Following the selection operation, a crossover operation is required to obtain additional chromosomes. Crossover refers to the exchange and recombination of certain structures of two-parent individuals to form a new individual. Common crossover operations include single-point crossover and multipoint crossover. Single-point crossover refers to the exchange of one gene, while multipoint crossover refers to the exchange of multiple genes simultaneously. To identify the optimal solution most efficiently, this paper employs both single-point crossover and multipoint crossover. Figure 4a shows the single-point crossover and Figure 4b shows the process of multi-point crossover.

**Figure 5:**
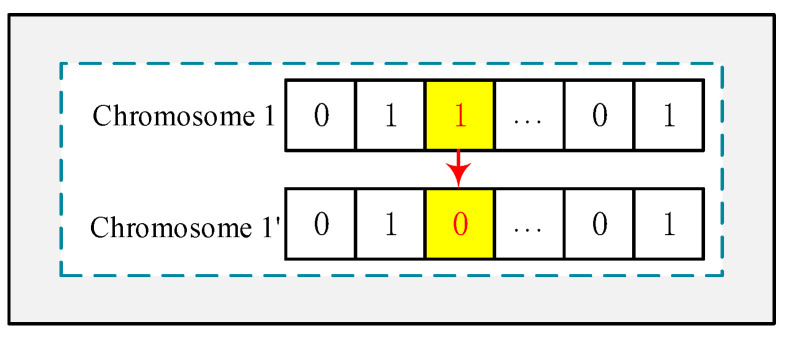
Mutation Operation.

**Mutation operation**: A mutation operation refers to the alteration of the position of specific genes within a population. In this paper, binary coding is employed, thus binary mutation is utilized. Figure 5 shows the process of mutation.

### 4.2. Urban POI Perception Algorithm Based on DBSCAN Clustering

In the urban POI sensing cluster model proposed in this paper, users upload data containing location information and a corresponding description. After evaluating various clustering algorithms and their suitability for different application scenarios, the DBSCAN algorithm is employed for the analysis of mobile cluster sensing data. Finally, get the accurate POI perception map for the city.

The basic definition of the DBSCAN algorithm:

1. **Neighborhood**: Given any sample point a, the area within the radius of Eps is called the neighborhood of the sample point a. Eps is called the neighborhood radius.
2. **Core point**: If the neighborhood of a given sample point a contains at least MinPts sample points, then point a is called a core point. MinPts is called the minimum number of points in the cluster.
3. **Density direct access**: For sample set D. If a sample point b is in the neighborhood of a, and a is the core point, then the sample points a to b are said to be a direct about the density of Eps and MinPts.
4. **Density reachable**: For a sample set D, given a series of sample points $a_{1},a_{2},\ldots ,a_{i},\ldots ,a_{n}$, ${a=a}_{i}$, ${b=a}_{i}$. If the sample point $a_{i}$ is directly accessible from $a_{i-1}$ density, then sample point a to sample point b is density reachable concerning Eps and MinPts.
5. **Density connection**: There is a point o in the sample set D, if the sample point o is density reachable to point a and point b, then a and b are density connected.

**Figure 6:**
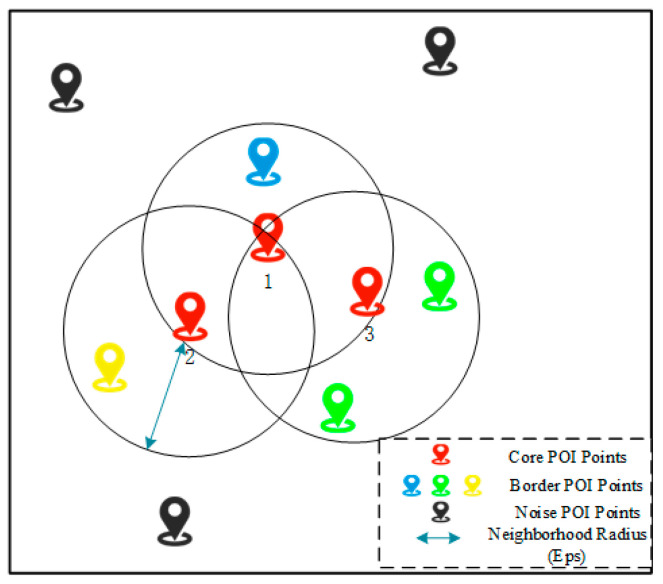
DBSCAN clustering example diagram.

To understand the above definition more clearly, Figure 6 shows a simple example of DBSCAN clustering on city location coordinate points. In this example, the minimum number of points in the cluster is set as Minpts = 3, and the neighborhood radius Eps is blue in Figure 6, indicated by the double arrow. In Figure 6, the red position coordinate points are the core POI coordinate points, the blue, yellow, and green position coordinate points are called boundary POI coordinate points, and the black position coordinate points are called noise POI coordinate points. The relationship between the blue position coordinate point and the core POI coordinate point 1 is density directness, the relationship with the other two core POI coordinate points is density reachability, and the relationship with the green and yellow position coordinate points is density connection. The density connection has symmetry. Therefore, the relationship between the yellow position coordinate point and the blue and green position coordinate points is also a density connection. Similarly, the relationship between the green position coordinate point and the blue and yellow position coordinate points is also a density connection. In urban POI perception, the purpose of the DBSCAN algorithm is to find the largest set of density-connected location coordinate points.

The clustering effect of the DBSCAN algorithm depends on the value of the neighborhood radius Eps and the minimum number of points in the cluster Minpts, but for different data dimensions and data volumes in different datasets, these two parameters are often difficult to determine. In the current study, the most common way to determine the parameters is to use the K-dist diagram [28]. The processing of using the K-dist graph to determine these two parameters is as follows: (1) Calculate the distance k-distance between each sample point in the dataset and its K-th neighbor. (2) Sort the k-distance of each sample point from large to small and draw a K-dist diagram. (3) The k-distance value with an obvious inflection point in the slope of the K-dist graph is taken as the neighborhood radius Eps, and the value of K is taken as the minimum number of cluster points Minpts.

In the above process of calculating the k-distance between two sample points, the traditional calculation method is to calculate the Euclidean distance between the two sample points. Euclidean distance represents the straight-line distance between two points in Euclidean space. Suppose the latitude and longitude coordinates of two sample points are A ($x_{1}$,$y_{1}$), B ($x_{2}$,$y_{2}$). Then the Euclidean distance d between them can be calculated by the following formula:

(1) $$\begin{array}{c} d=\sqrt{{\left(x_{1}-x_{2}\right)}^{2}+{\left(y_{1}-y_{2}\right)}^{2}}. \end{array}$$

However, Euclidean distance often cannot accurately reflect the real distance between two location coordinates, so in this paper, we use Haversine distance to represent the distance between two locations. The Haversine distance formula is a formula for calculating the distance between geospatial coordinate points based on a spherical model. Using this method to calculate the distance between two location coordinates on the earth can more accurately reflect the real distance between the two locations. For two sets of longitude and latitude coordinates, A ($x_{1}$,$y_{1}$) and B ($x_{2}$,$y_{2}$), the Haversine distance D between them can be calculated by the following formula:

(2) $$\begin{array}{c} D=2r\;*\\ \arcsin\sqrt{\sin^{2}\left(\frac{y_{1}-y_{2}}{2}\right)+\cos y_{1}\cos y_{2}*\sin^{2}\left(\frac{x_{1}-x_{2}}{2}\right)}, \end{array}$$

where r is the radius of the Earth 6371 km, and $x_{1},x_{2},y_{1},y_{2}$ are the radian values.

Based on the comparison of two types of distance calculation, we propose an improved density-based spatial clustering algorithm for noise applications (DBSCAN-H) by replacing the Euclidean distance in the traditional clustering algorithm with the Haversine distance. This improved method can more accurately deal with the problem of distance calculation in geospatial data, thus enhancing the performance of clustering algorithms in dealing with applications such as geographic information systems (GIS). Consists of the following steps of the DBSCAN-H algorithm used in this paper as follows: (1) Calculate the Haversine distance between each position coordinate points in the MCS fuse dataset and its K-th neighbor point as the k-distance of the position coordinate point. (2) Sort the k-distance and draw the K-dist diagram to determine the parameters of the DBSCAN-H algorithm, the neighborhood radius Eps, and the minimum number of cluster points Minpts. (3) After determining the parameters of the DBSCAN-H algorithm, the number of clusters is initialized to 0 and an unprocessed position coordinate point a is selected from the MCS fuse dataset. If the number of sample points contained in the Eps neighborhood of this point is not less than Minpts, the sample Point a is used as the core POI coordinate point, a cluster is created with point a as the core, and the sample points in the Eps neighborhood that are directly connected to point a are added to the cluster. (4) Add the position coordinate points that are density-reachable to all core POI coordinate points in the cluster. Continue adding until all position coordinate points that are density-connected to point a are included in the cluster. (5) Select a position coordinate point that has not been added to any cluster, and repeat the above process until no new position coordinate point can be added to any cluster. The clustering algorithm ends, and the position coordinate point that has not been added to any cluster is the noise POI coordinate point. Algorithm 1 presents the complete pseudocode for clustering the MCS fuse dataset using our proposed DBSCAN-H algorithm.
**Algorithm 1.** POI Perception Based on DBSCAN-H**Input**: Collection of sample points D. **Output**: Clustering result C.
1:  Number_C = 0//Initialize the number of clusters to 0
2:  Compute the k-distance for each sample point to obtain the neighborhood radius Eps.
3:  Calculate the average expectation of the number of sample points within all neighborhood radii to obtain the minimum number of points in the cluster, MinPts.
4:  **for** each unvisited point p in the sample set D **do**
5:      Mark p as visited
6:      Compute the number of sample points N within the neighborhood radius Eps of point p
7:      **if** N < MinPts **then**
8:          Mark p as a noise point
9:      **else**
10:         Create a new cluster C
11:         Add p to cluster C
12:         **for** each unvisited sample point $p^{\prime }$ within p **do**
13:             Mark $p^{\prime }$ as visited
14:             Compute $N^{\prime }$ within Eps of point $p^{\prime }$
15:             **if** $N^{\prime }$ > Minpts **then**
16:                 $\;N\;=\;N\;+\;N^{\prime }$
17:             end if
18:             **if** $p^{\prime }$ does not belong to any other cluster then
19:                 Add $p^{\prime }$ to cluster C
20:             end if
21:         end for
22:     end if
23: end for
24: return C

## 5. Experiments and Results

All experiments in this chapter were conducted on a Windows 11 operating system, running on an Intel(R) Core (TM) i5-12490F CPU (Intel Corporation, Santa Clara, CA, USA) with 8 GB of RAM. The programming language used is Python 3.8, and the experimental platform employed is Pycharm 2020.1.6. This information enables replication of the experimental setup. To test the efficiency of the genetic algorithm-based worker recruitment algorithms proposed in this chapter, we perform 100 experiments for each algorithm to eliminate any experimental coincidence. The average of the results from the 100 experiments was ultimately taken as the outcome. To validate the efficacy of the proposed methodology, multiple experiments are conducted on both real simulation datasets and open-source datasets. The following sections will present DBSCAN-H, and MCS-APF experimental results and analysis.

### 5.1. WR-GA Experimental Result and Analysis

#### 5.1.1. Experimental Parameter Setup

As part of the effort to recruit skilled workers capable of providing quality sensory data, this chapter aims to test the effectiveness of the proposed genetic algorithm. To achieve this, a simulation dataset was created by randomly generating workers within a fixed POI point radius and setting their relevant genera values. Table 2 details the parameters of the recruitment experiments conducted on this dataset.

#### 5.1.2. Baseline Algorithm

(a) Quality Greedy Recruitment Algorithm (Q-Greedy). The processing of this algorithm is to recruit workers who can provide maximum data quality until the budget of the sensing platform is exhausted.
(b) Quantitative Greedy Recruitment Algorithm (N-Greedy). The algorithm proceeds to recruit the workers that require the least amount of compensation until the perceived platform budget is depleted.
(c) Traditional Recruitment Algorithm (Traditional). This algorithm uses a traditional algorithm with criteria for recruiting workers until the budget is exhausted, given a certain budget for the perceptual platform.

#### 5.1.3. Evaluation of Indicators

(a) Sum of Perceived Data Quality. The sum of perceived data quality values represents the total quality of data that the platform can submit from several recruited workers. This metric can be calculated by,
  (3) $$\begin{array}{c} Q_{total}=\sum_{i=1}^{N}{sig}_{i}\left(\varepsilon_{i}^{t}*r_{i}^{t}*h_{i}^{t}+\gamma Q_{i}^{t-1}\right). \end{array}$$
(b) Average perceived data quality. The average perceived data quality $Q_{average}$ refers to the sum of the quality of the data that can be submitted by several workers after they have been recruited by the perception platform, divided by the number of workers N
  (4) $$\begin{array}{c} Q_{average}=Q_{total}/N. \end{array}$$

#### 5.1.4. Analysis of the Results of WR-GA

In this section, we examine the experimental outcomes of the worker recruitment algorithm utilizing the genetic algorithms suggested in this paper, in contrast with other benchmark algorithms.

**Figure 7:**
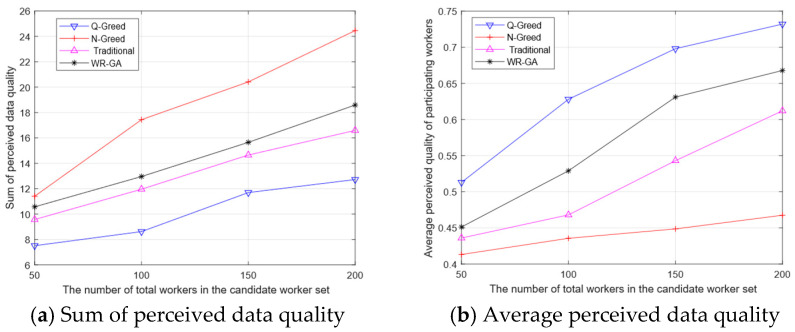
Impact of the total number of workers.

Figure 7a shows the effect of the number of total workers in the candidate set on the sum of perceived data when the number of workers is fixed. The sum of perceived data quality obtained by the WR-GA algorithm is significantly higher than that of the Q-Greedy and traditional algorithms. The WR-GA algorithm, through the cross-variance operation, considers both the quality of the data submitted by the workers and the compensation they demand. By reasonably measuring both factors, it recruits more workers who can provide high-quality data at a lower price. The N-greedy algorithm can obtain the highest sum of perceptual data quality. However, it does so by focusing on quantity and gathering a large amount of low-quality data. As a result, despite achieving a high data quality sum, the algorithm falls short in terms of average data quality. This deficiency prevents the data from providing an accurate perception of urban POI. As can be seen from Figure 7, the WR-GA algorithm achieves a 15% improvement in the sum of perceived data quality compared to the traditional algorithm.

Figure 7b shows the effect of the number of total workers in the candidate set on the average number of perceptions: as the total number of workers increases, the percentage of workers able to provide high-quality data increases, and so does the average quality of the perceived data. Although the Q-Greedy algorithm acquires the highest average quality of perceived data, it is a quality-greedy algorithm. Even though it acquires the highest quality data, the amount of data it acquires is not sufficient to support POI perception. The WR-GA algorithm acquires a significantly higher average quality than the N-Greedy algorithm and traditional algorithms, because the WR-GA algorithm, considering the proposed criteria incorporating the spatiotemporal characteristics of the workers, can acquire high-quality data within the cost constraints. As can be seen from Figure 7b, the WR-GA algorithm improves the quality of the average perceptual data obtained by 10% compared to the traditional algorithm.

**Figure 8:**
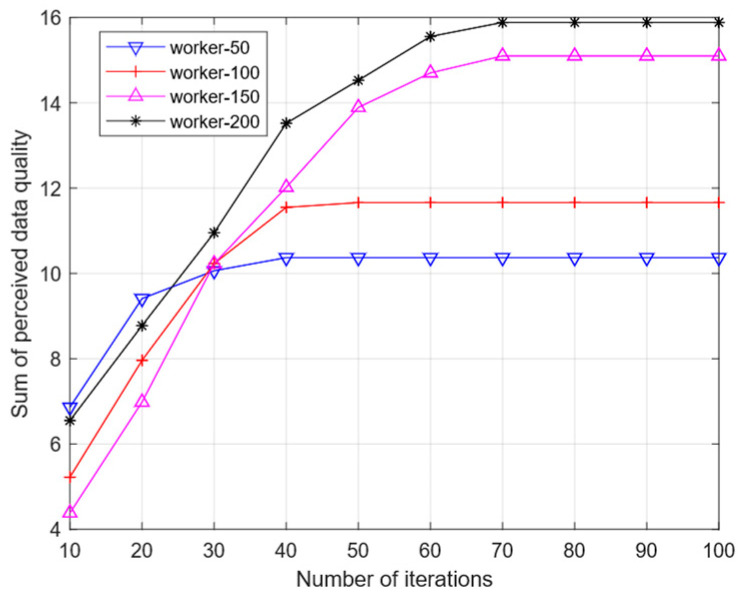
Effect of the number of iterations on the sum of perceived data quality.

Figure 8 illustrates the impact of iteration count on the overall perceived data quality when utilizing the WR-GA algorithm to solve for the optimal solution. It is evident from the graph that, as the total number of workers remains constant, the sum of perceptual data quality increases with more iterations. Eventually, the curve flattens out once the number of iterations reaches a certain threshold, implying that the solution has converged to optimality. When the graph is observed vertically, it becomes apparent that as the number of iterations remains constant, the greater the total number of workers, the higher the total sum of perceived data quality. This is because an increase in the total number of workers results in a rise in the proportion of those who can provide high-quality data, allowing the algorithm to recruit such workers and thus enhancing the overall sum of perceived data quality.

### 5.2. DBSCAN-H Experimental Result and Analysis

#### 5.2.1. Parameter Setup

For the investigation into the detection of urban POI based on mobile swarm perception data, we used the Foursquare dataset [33]. This dataset was selected as the experimental data source based on several considerations. First, the dataset contains a large-scale collection of urban check-in records (comprising 227,428 check-ins), which adequately validates algorithm performance under big data scenarios. Second, the dataset comprehensively covers the entire Tokyo area, with broad geographical distribution and representativeness, making it suitable for urban POI distribution analysis. Third, the data encompasses eight major categories of points of interest (including catering, medical facilities, transportation hubs, etc.), providing rich diversity that enables comprehensive evaluation of classification effectiveness across different POI types. Fourth, the reasonable temporal span (April 2012 to February 2013) avoids excessive influence of seasonal factors on POI distribution patterns. Finally, the Foursquare dataset has been widely adopted in location-based services and social sensing computing research [34], offering good comparability and reproducibility that facilitates comparative analysis with other research outcomes. The comprehensive details of this dataset are presented in Table 3. After removing duplicates, erroneous data, and instances with missing values, a total of 4998 entries about food and beverage establishments were identified.

#### 5.2.2. Baseline Algorithms

1. Traditional DBSCAN algorithm [29] (DBSCAN-E): This algorithm aims to analyze the business structure characteristics of a city by using the DBCAN algorithm on its restaurant and shopping POI data. The algorithm identifies domain moves and cluster minima in the following manner:
  (5) $$\begin{array}{c} Eps=\sum_{k=1}^{20}{mean}_{i}/k/n, \end{array}$$
  where ${mean}_{i}$ represents the average sum of distances between nearby POIs surrounding the ith central point, k indicates the count of neighboring POIs nearest to the central point, and n stands for the total POI count. Once the Eps parameter is determined, the minimum number of points required for clustering is calculated as the expected value of the number of points within the domain radius of each point.
  (6) $$\begin{array}{c} Minpts=\frac{1}{n}\sum_{i=1}^{n}{count}_{i}n, \end{array}$$
  where ${count}_{i}n$ is the number of sample points in the domain of sample points.
2. K-means clustering algorithm [27]: K-means clustering is an unsupervised learning and partitioning algorithm used for clustering. It involves dividing a collection of samples into k subsets, which in turn constitute k classes. This study uses the K-means algorithm to perform cluster analysis of POI data associated with metro stations for fine-grained categorization of the stations.
3. HDBSCAN algorithm [26]: As an advanced density-based algorithm, HDBSCAN is known for its ability to find clusters of varying densities without requiring the Eps parameter. We used the hdbscan Python library with its default parameters, setting min-cluster-size to 15 to ensure a fair comparison with the Minpts parameter used in DBSCAN-based methods.

#### 5.2.3. Evaluation of Indicators

1. CH Index: The CH index (Calinski–Harabaz Index) quantifies the closeness of a class by computing the sum of the squares of the distances between each point within the class and its center, as well as the separation of the dataset by computing the sum of the squares of the distances between each class center and the center of the entire dataset. The value of the CH index is then obtained by dividing the separation measure by the closeness measure. A higher CH index indicates that the class is more tightly clustered and less dispersed, resulting in better clustering outcomes. Here is the specific representation of the CH index:
  (7) $$\begin{array}{c} CH=\frac{tr\left(B_{k}\right)}{tr\left(W_{k}\right)}*\frac{N_{E}-k}{k-1}, \end{array}$$
  where $N_{E}$ denotes the number of training samples in the dataset, k denotes the number of categories, $B_{k}$ denotes the covariance matrix between categories, $W_{k}$ denotes the covariance matrix between sample points within a class, and tr(·) denotes the trace of a matrix.
2. DBI Index: The DBI index (Davies–Bouldin index), also known as the Classification Accuracy Index, is a significant measure utilized for evaluating the capabilities and limitations of clustering algorithms. The Davies–Bouldin index (DBI) is computed by dividing the average sum of the distances between pairs of objects within a cluster by the distance separating the cluster centers, and selecting the maximum value. The smaller the resulting DBI value, the better the clustering performance in terms of the smaller intra-cluster distance and larger inter-cluster distance. The formula for the DBI is as follows:
  (8) $$\begin{array}{c} DBI=\frac{1}{k}\sum_{1}^{k}\frac{s_{i}+s_{j}}{d_{i,j}},i\neq j, \end{array}$$
  where $s_{i}$ denotes the average distance of each point in class i from the center of mass of that class, and $d_{i,j}$ denotes the distance between class i and the center of mass of class *j*.

The selection of CH and DBI indices as clustering performance evaluation metrics is well justified. The CH index comprehensively reflects cluster compactness and separation by calculating the ratio between the trace of the between-cluster dispersion matrix and the within-cluster dispersion matrix. This is particularly suitable for evaluating the aggregation characteristics of POI distributions in geospatial data—a high CH value indicates that POIs form well-defined spatial aggregation patterns while maintaining clear separation between different categories. The DBI, conversely, assesses clustering quality from a complementary perspective by computing the average maximum similarity between clusters to measure both intra-cluster consistency and inter-cluster distinction. A low DBI value signifies high consistency within POI categories and clear differentiation between them, which directly reflects the accuracy of POI classification. The combined use of these two metrics enables a comprehensive evaluation of clustering algorithm performance in POI perception tasks from complementary angles, considering both the macroscopic characteristics of spatial distribution and the microscopic quality of category.

#### 5.2.4. Analysis of the Results of DBSCAN-H

**Figure 9:**
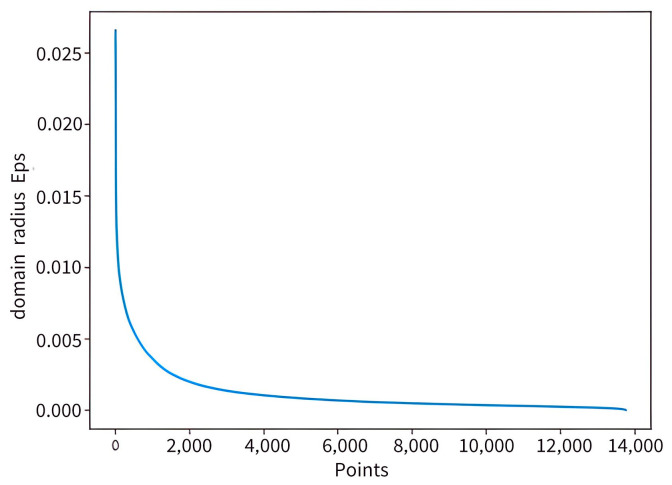
Haversine distance-based K-dist plot.

The two key parameters of the clustering algorithm, namely domain radius and minimum number of cluster points, exhibit some variation across different datasets. This section employs the K-dist plot to ascertain the domain radius and minimum number of clusters for the DBSACN-H algorithm on the Foursquare dataset. With K = 4, the figure presents the K-dist plot of the DBSCAN algorithm on the Foursquare dataset. Figure 9 provides a detailed illustration indicating that the domain radius Eps of the Foursquare dataset is within the range of [0.2, 0.4] in the event of explicit inflection points on the slope of the K-dist plot. For certain experiments, the domain radius Eps for this dataset is established as 0.04.

**Figure 10:**
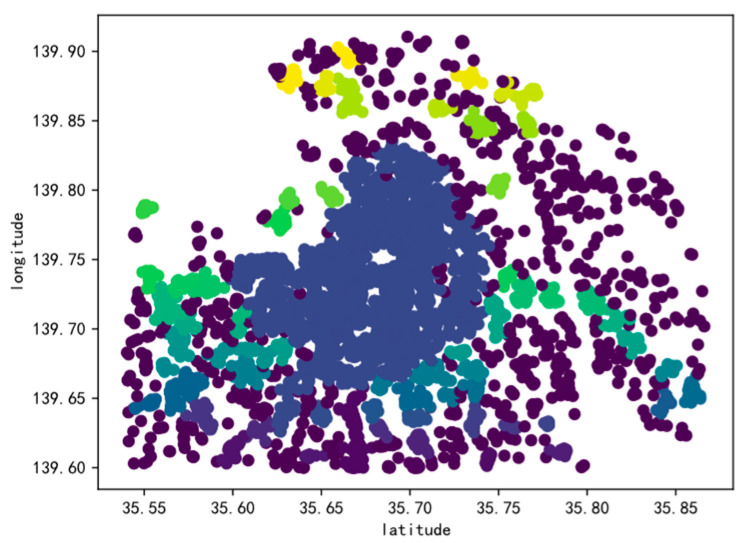
DBSCAN-H of Clustering results.

Using the DBSCAN-H algorithm advanced in this paper, all the sample points from the Foursquare dataset have been identified into 11 distinct categories, along with 41 noise points. The outcomes of the clustering done on this dataset are exhibited in Figure 10.

**Figure 11:**
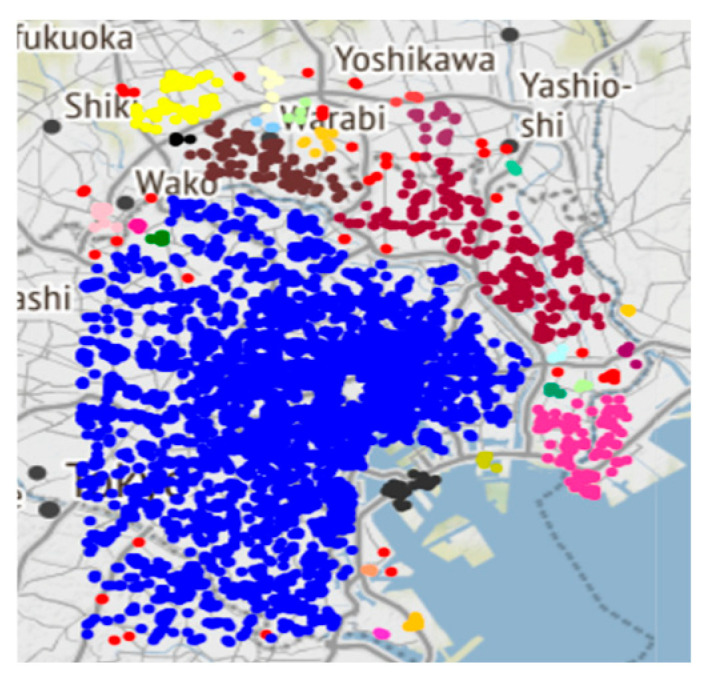
Tokyo Real Map Clustering results.

Figure 11 depicts the clustering scenario on an actual map of Tokyo, revealing a prosperous restaurant industry that spans the city’s limits and possesses significant concentrations.

**Figure 12:**
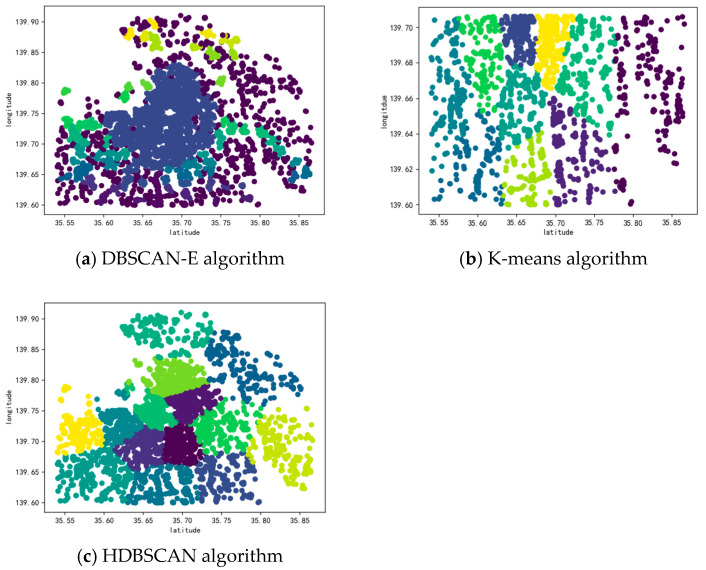
Results of Baseline Algorithms.

Figure 12 shows clustering results using DBSCAN-E, K-means clustering and HDBSCAN on the Foursquare dataset. Specifically, the DBSCAN-E algorithm employs a domain Eps of 0.6 and a minimum number of points in clusters, MinPts of 16, which are calculated by Equations (5) and (6). The K-means algorithm utilizes the same number of clusters, K, as those in the clustering outcomes of the DBSCAN-H algorithm, and both are 14. As displayed in Figure 12a, clustering using the DBSCAN-E algorithm produces six clusters, which is notably fewer than the number of clusters generated by the DBSCAN-H algorithm in Figure 10. Some of the sample points can be grouped into different clusters, but due to the Haversine distance calculation between their geographic locations being more accurate in reflecting the real distance between them, some are classified into the same cluster. When comparing Figure 10 and Figure 12b, it becomes evident that the K-means clustering algorithm is less effective in the Foursquare dataset, as numerous sample points belonging to the same class are inconsistently clustered into different clusters. The comparison between Figure 12c and Figure 10 reveals that while both algorithms can handle clusters of varying densities, DBSCAN-H achieves better cluster cohesion and separation, particularly in complex urban environments with mixed POI distributions. This demonstrates the effectiveness of our approach over traditional density-based clustering methods.

The results presented in Table 4 demonstrate that our proposed DBSCAN-H algorithm outperforms all baseline algorithms, including the advanced HDBSCAN, in terms of both the CH Index and DBI. DBSCAN-H achieves the highest CH value, indicating that its clustering results possess the best intra-cluster compactness and inter-cluster separation. Concurrently, its lowest DBI value further confirms that it maintains smaller intra-cluster distances while achieving larger inter-cluster distances.

Specifically, examining the performance of the HDBSCAN algorithm (CH: 15,200,000.000, DBI: 0.01500), which is renowned for its ability to handle varying densities without requiring the Eps parameter, reveals that it is outperformed by DBSCAN-H in this specific task. We posit that this is because HDBSCAN’s hierarchical construction and cluster extraction based on stability can sometimes be overly conservative. In our POI dataset, this may lead to the misclassification of lower-density peripheral points belonging to a valid POI as noise, or the spurious splitting of a cohesive cluster into smaller, less significant ones. This behavior can undermine the goal of achieving high intra-cluster compactness for POI perception. In contrast, DBSCAN-H, leveraging a carefully selected global Eps parameter, more consistently captures geographically proximate POI aggregations, forming clusters that better align with our perception requirements for distinct urban points of interest.

### 5.3. MCS-APF Perception Result and Analysis

#### 5.3.1. Parameter Setup

Due to factors such as personal privacy protection and the experimental environment, this paper adopts a simulation-based experimental approach to approximate different quality and quantity of datasets obtained by the WR-GA algorithm, as shown in the simulation results in Section 5.3.1. To simulate the quantitative impact, we randomly remove parts of the dataset according to a certain experimental ratio to generate results. To simulate the quantity impact, the typical noise processing methods usually consist of two types: adding random offsets and data perturbation. Random offsets introduce randomness by adding random numbers generated from a normal or uniform distribution to each coordinate value, thereby controlling the amount of noise and adjusting the uncertainty of the data. Data perturbation, on the other hand, slightly alters the data by multiplying each coordinate value by a random number close to 1 or by adding small random values, thus adding randomness while maintaining the original coordinate relative positional relationships, making the data more realistic and representative.

To better simulate quality impacts and generate more diverse and realistic datasets, in this paper, we designed a data processing scheme, called the Combined Perturbation and Offset Method (CPOM), that combines both of the above two methods. First, we processed the coordinates, time, and heat attributes of the POI in the original dataset with random offsets. Then, small perturbations are applied to the processed data to further increase the randomness while maintaining the relative positional relationships of the original data. This method aims to construct noise conditions that are closer to real-world environments, thereby testing the robustness of the proposed MCS-APF framework under complex scenarios.

**Figure 13:**
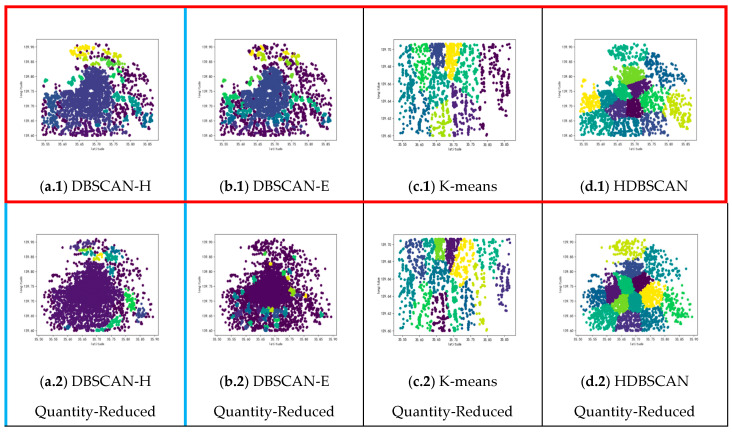

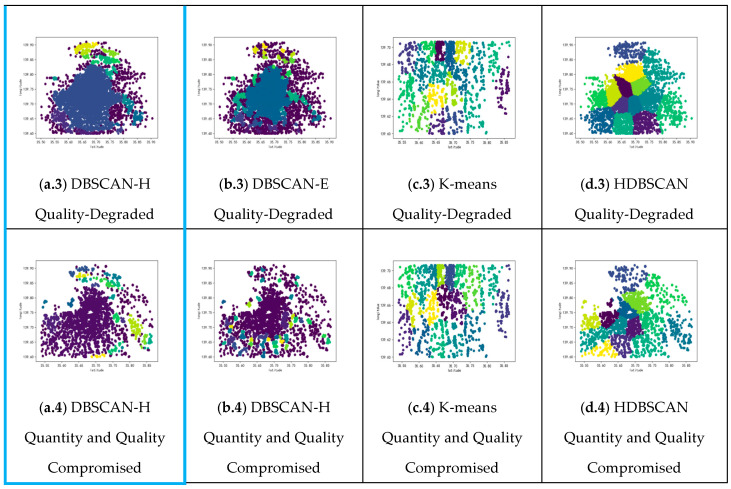
Clustering results of different clustering algorithms in different datasets.

The data used for the experiments in this chapter are divided into four datasets: (1) the complete dataset; (2) the reduced quantity dataset; (3) the degraded quality dataset, and (4) the quantitatively and qualitatively impaired dataset. The results of the same algorithm with four datasets are represented sequentially as columns in Figure 13. In details, (1) is the Foursquare dataset, and (2) is obtained by randomly deleting 20% of the data based on (1). (3) is obtained by CPOM scrambling based on (1), where CPOM sets the standard deviation of noise to 0.025, the proportion of noise to 0.3, and the attributes of noise are coordinates, time, and heat among the seven attributes of the Foursquare dataset. (4) is obtained by randomly deleting 20% of the data based on (3).

#### 5.3.2. Analysis of the Results of MCS-APF

Four clustering algorithms, DBSCAN-H, DBSCAN-E, K-means and HDBSCAN, are used in this chapter, and experiments are conducted on four different datasets. The experimental results are shown in Figure 13.

In the first column of Figure 13, as shown in the blue box, we compare the performance of datasets (1)–(4) using DBSCAN-H simulation. By pairwise comparison of the clustering results, we can assess the impact of different perceptual data acquisition algorithms on the quantity and quality of datasets under identical conditions. The clustering outcomes illustrated in Figure 13a.1–a.4 demonstrate that the datasets (1) exhibit superior compactness within the clustering space, with denser data points and more distinct separations between clusters, compared to the other datasets. This indicates that the WR-GA algorithm produces higher-quality datasets, thereby enhancing both the quantity and quality of the data obtained. Additionally, the WR-GA algorithm generates a larger number of high-quality perceptual data points, further increasing the overall dataset quantity. These findings confirm the superiority of the WR-GA algorithm in recruiting workers and acquiring perceptual datasets. In conclusion, our experiments provide strong theoretical and empirical evidence that the WR-GA algorithm outperforms traditional recruitment algorithms in both the quality and quantity of perceptual datasets, supporting its application in the subsequent perception of POI through worker recruitment.

In each row of Figure 13, as shown in the red box, we compare the performance of the clustering results of the four clustering algorithms under the same dataset. The different rows show the comparisons of the different algorithms with the four datasets. It can be seen that the clustering results obtained using the DBSCAN-H algorithm proposed in this paper outperform the other algorithm, demonstrating higher accuracy and better clustering results. While the quality and quantity of the dataset are enhanced with the assistance of WR-GA, DBSCAN-H improves the performance of perceptual clustering results more significantly compared to other algorithms.

Comparing Figure 13a.1 with the other subfigures, it is evident that MCS-APF not only enhances the quality and quantity of the dataset but also yields more accurate POI results through the clustering process. The results indicate that the POI classification by the MCS-APF is superior in accuracy, speed, and cost-effectiveness compared to the traditional POI classification method. This demonstrates the overall effectiveness of the MCS-APF in improving the accuracy and efficiency of POI classification.

## 6. Conclusions

In conclusion, the MCS-APF framework presented in this paper represents a significant advancement in urban POI classification. By integrating a novel worker recruitment criterion and the WR-GA algorithm into the data collection module, the framework has achieved a notable increase in both data quantity and quality. The DBSCAN-H algorithm proposed in the clustering module, utilizing a Haversine distance-based approach, has further enhanced the accuracy of POI classification. The experimental results obtained from MCS-APF highlight its superiority in providing rapid, cost-effective, and precise urban POI sensing, which is highly relevant for urban planning and development.

Looking ahead, our future work will focus on two primary directions: first, enhancing the adaptability of the WR-GA algorithm for dynamic environments and real-time scenarios; second, validating and extending the DBSCAN-H algorithm across multiple cities and diverse geographical contexts to strengthen the generalizability of the proposed framework. We aim to improve the data collection algorithms and clustering methods to enhance the accuracy and timeliness of the sensed data, thereby creating more opportunities for sensing and analyzing POIs in urban areas.

## Figures and Tables

**Table 1:** Comparison of Existing POI Perception Approaches and Identification of Research Gaps.

Approach Category	Key Focus	Limitations/Identified Gaps	How MCS-APF Addresses the Gap
Data Fusion Methods [6,8,9,11,12]	Integrating multi-source data (e.g., photos, check-ins) to improve POI coverage and description.	Often neglects the cost-quality trade-off in data acquisition. Assumes availability of high-quality user-generated content.	**Hybrid Sensing & WR-GA:** Balances cost and quality by fusing limited high-quality participatory data with large-scale, low-cost opportunistic data. WR-GA ensures quality under a budget.
Clustering-Based Techniques [7,13,27,28,29,30,31,32]	Using spatial clustering algorithms (e.g., DBSCAN variants) to identify POIs from geographic data.	Spatial Inaccuracy: Often uses Euclidean distance, unsuitable for geospatial data. Data Dependency: Performance relies on preprocessed, high-quality input data.	**DBSCAN-H Clustering Algorithm:** Employs Haversine distance for accurate geospatial clustering. The framework’s data collection ensures robust input.
MCS Frameworks [17,18,19,20,21]	Leveraging mobile users for scalable data collection via task assignments and incentives.	Worker Recruitment: Fails to recruit high-quality workers under budget constraints. Sensing Paradigm: Often uses only one paradigm (participatory or opportunistic), limiting data scope.	**WR-GA Algorithm & Hybrid Sensing:** Optimizes worker recruitment for data quality within a budget. Integrates both sensing paradigms to maximize coverage and quality.
Our Work: MCS-APF	An integrated framework for accurate, cost-effective, and scalable urban POI classification.	**Target:** To solve the trilemma of achieving High Data Quality, Low Cost, and High Clustering Accuracy simultaneously.	**Holistic Solution:** Integrates a hybrid sensing strategy, a quality-aware recruitment algorithm (WR-GA), and a spatially accurate clustering algorithm (DBSCAN-H).

**Table 2:** Experimental parameters for worker recruitment.

Parametric	Value
Total number of workers N	[50, 100, 150, 200]
Total platform budget B	100
Worker capacity *ε*	0–1
Worker credibility R	0–1

**Table 3:** Foursquare Dataset Details.

Type of Checked-in Place	Data Volume
Catering	60,650
Medical Category	5907
Transport Hubs	25,136
Science and Education	8420
Shopping mall consumer	17,215
Office space category	22,729
Sports and Leisure	22,362
Others	65,009

**Table 4:** Comparison of CH and DBI values of different algorithms.

Algorithms	CH	DBI
DBSCAN-H	27,768,483.118	0.01099
DBSCAN-E	1,370,026.845	0.01941
K-means	10,099.244	0.75512
HDBSCAN	15,200,000.000	0.01500

## Data Availability

Data are contained within the article. The Foursquare dataset used in this study is publicly accessible through standard academic data repositories.
